# Serotonylation: A novel histone H3 marker

**DOI:** 10.1038/s41392-019-0048-7

**Published:** 2019-05-10

**Authors:** Lin Fu, Lingqiang Zhang

**Affiliations:** 1 Institute of Chronic Disease, Qingdao Municipal Hospital, Qingdao University, 266000 Qingdao, China; 2 State Key Laboratory of Proteomics, National Center of Protein Sciences (Beijing), Beijing Institute of Lifeomics, 100850 Beijing, China

**Keywords:** Epigenetics, Biochemistry

A recent study by Farrelly, L.A*.* et al. published in Nature showed that the serotonylation of glutamine at the Q5 site in histone H3 promotes the recruitment of TFIID in collaboration with adjacent H3K4 trimethylation^1^.

Protein posttranslational modifications (PTMs) of histones are associated with diverse biological processes through dynamically modulating chromatin structure and the transcriptional activity of genes^2^. Epigenetic regulations are an integral part of brain functions ranging from the development of the nervous system beyond basic neurological functions to higher-order cognitive processes^3^.

Serotonin, also known as 5-hydroxytryptamine (5-HT), is an excitatory neurotransmitter that regulates brain cell activity by activating specific membrane receptors in the brain^4^. The amino group of serotonins can covalently bind to the glutamine-ã-carboxamide of cytosolic proteins^5^. This kind of transamination reaction is called serotonylation. Interestingly, as a small extranuclear biological molecule, serotonin can also enter the nucleus while exerting its function as a neurotransmitter^6^.

Recently, Farrelly et al. revealed that histone H3 but not histones H2A, H2B, and H4 was modified with serotonin^1^. This is the first identified endogenous monoamination modification of histone and the first reported modification of a glutamine residue on a nonmethylated histone.

Transglutaminase 2, tissue transglutaminase 2 (TGM2) can directly attach serotonin molecules to nonhistone proteins^7^. In addition, serotonin can crosslink histone proteins in vitro^8^. Farrelly et al. observed the attachment of serotonin to the glutamine residue at the fifth position of histone H3 by TGM2 to form histone 5-hydroxytryptamine (H3Q5ser)^1^. Almost all PTMs have unique regulatory enzyme profiles. Thus, apart from transglutaminase, whether any demonoaminylases participate in this process requires further investigation.

H3K4 trimethylation (H3K4me3) is a critical marker of active transcription, enhancer signatures, and developmentally stable genes^9,10^. Because H3Q5ser modifies sites adjacent to H3K4me3, crosstalk might exist. However, H3K4me3 modification did not affect the activity of H3Q5ser produced by TGM2 in vitro, and H3Q5ser modification did not affect the activity of H3K4me3 produced by MLL1 in vitro. Moreover, the H3K4me3Q5ser signal was broadly distributed in nerve, heart, and testicular tissues and peripheral blood mononuclear cells. Interestingly, even nonserotonergic neurons and nonneuronal cells had a strong H3K4me3Q5ser signal^1^. Transporters such as the 5-HT transporters (SERTs), the organic cation transporters (OCTs), and the plasma membrane monoamine transporters (PMATs) can shuttle serotonin into cells^7^. Further research is needed to identify the essential transporter involved in this process.

The presence of H3K4me3Q5ser enhanced the binding of transcription factors, such as TFIID to chromatin, thereby activating the expression of downstream genes in developing rodent brains and human neurons. Furthermore, H3K4me3Q5ser was enriched in the promoter regions of genes related to neuronal differentiation, such as ELAVL3^1^. In addition to its role in differentiation, serotonin regulates mood, affection, cognition, aggression, satiety, sleep and other autonomic functions^3^. Does serotonylation also play a role in these processes? Can this new knowledge be used to develop better medications to treat related diseases?

Serotonin is only one of the many in vivo neurotransmitters with amino groups. Monoamine neurotransmitters such as norepinephrine, dopamine, and histamine monoaminylate substrates^7^. How do these various monoamines and more similar modifications participate in the regulation of epigenetic and gene expression?

**Fig. 1:**
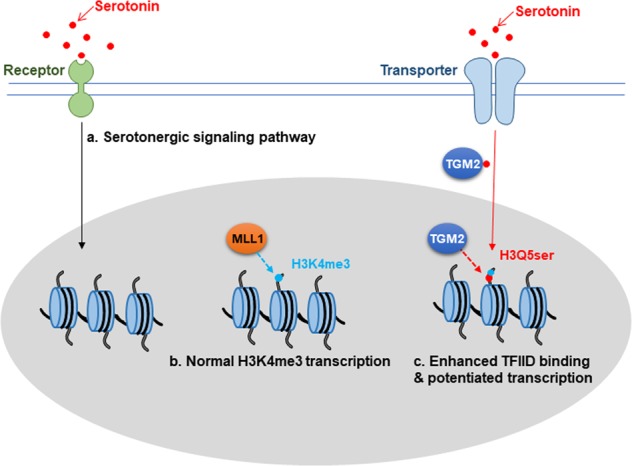
Regulation model of serotonin. **a** Serotonin, a neurotransmitter, functions through the serotonergic signaling pathway. **b** MLL and SET1 mediate H3K4me3 methylation and regulate gene expression. **c** The H3K4me3Q5ser double modification enhances the interaction of histone with certain H3K4me3 ‘reader’ proteins, such as TFIID, and then reinforces permissive patterns of gene expression

In other words, serotonin is more than a neurotransmitter. On the one hand, serotonin activates intracellular signal transduction pathways through cell surface receptors to influence intracellular biochemical changes. On the other hand, serotonin is transported into the cell and finally into the nucleus through cell surface transporters. It is then conjugated to H3K4me3-marked nucleosomes to form H3K4me3Q5ser, which enhances TFIID binding and changes gene expression patterns (Fig. 1).

In view of the roles of serotonin in diverse cellular processes, this study not only opens the opportunity to reveal unknown cellular mechanisms controlled by serotonin but also sheds new light on the epigenetic roles of serotonylation. This study leaves us with certain questions worth being investigated. The functions of the single-site modification H3Q5ser still require further study. In addition, whether other specific H3K4me3Q5ser ‘readers’ exist under different physiological conditions is unknown. What is the ratio of the H3K4me3Q5ser double modification to the H3K4me3 single modification in vivo? What is the role of H3K4me3Q5ser in tissues other than the brain?

Although this work has led to a series of new questions that need to be answered, it extends the function of serotonin in epigenetic fields and suggests other possible monoamine modifications. These novel functions may significantly contribute to our understanding of related diseases and propose new therapeutic targets.
